# Dual-drug nanocarriers for gout therapy: targeted co-delivery of anti-inflammatory and urate-lowering agents: a review

**DOI:** 10.17179/excli2025-8871

**Published:** 2025-11-13

**Authors:** Nisha Rata Karusan, Hairul Anuar Tajuddin, Nor Azlin Mat Radi, Rumman Karimah, Pratiwi Soesilawati, Syed Mahmood, Noraini Ahmad

**Affiliations:** 1 Department of Chemistry, Faculty of Science, Universiti Malaya, 50603 Kuala Lumpur, Malaysia; 2 Centre for Fundamental and Frontier Sciences in Nanostructure Self-Assembly (FSSA), Department of Chemistry, Faculty of Science, Universiti Malaya, 50603 Kuala Lumpur, Malaysia; 3 Universiti Malaya Community Engagement Centre (UMCares), Universiti Malaya, 50603 Kuala Lumpur, Malaysia; 4 Undergraduate Medical Study Program, Faculty of Medicine and Health, Institut Teknologi Sepuluh Nopember, Surabaya, East Java 60111, Indonesia; 5 Department of Oral Biology, Faculty of Dentistry, Universitas Airlangga, Surabaya, East Java 60132, Indonesia; 6 Department of Pharmaceutical Technology, Faculty of Pharmacy, Universiti Malaya, 50603 Kuala Lumpur, Malaysia; 7 Faculty of Pharmaceutical Sciences, Chulalongkorn University, Pathum Wan, Bangkok, 10330, Thailand

**Keywords:** gout, monosodium urate, nanocarrier, niosomes, anti-inflammatory, allopurinol

## Abstract

Gout, a prevalent form of inflammatory arthritis, arises from the deposition of monosodium urate crystals in joints due to chronic hyperuricemia. Current pharmacologic monotherapies such as xanthine oxidase inhibitors, uricosurics, NSAIDs, corticosteroids, and colchicine are often limited by inadequate dual-action efficacy, suboptimal bioavailability, and systemic side effects. Emerging nanocarrier-based drug delivery systems offer a promising alternative by improving pharmacokinetics and enhancing targeted delivery to inflamed tissues. While co-encapsulation of multiple therapeutics remains underexplored in gout, advances in related inflammatory diseases support its future application. This review explores the limitations of conventional gout therapies and highlights recent advancements in nanocarrier technologies, including liposomes, niosomes, and ethosomes, for delivering both anti-inflammatory and urate-lowering agents. Special attention is given to functionalization strategies that allow for site-specific delivery and sequential drug release, particularly in the acidic and oxidative microenvironments characteristic of acute gout flares. Co-delivery of agents such as allopurinol or febuxostat with NSAIDs or corticosteroids may reduce pill burden, improve therapeutic synergy, and enhance patient adherence. While clinical translation remains in early stages, the mechanistic rationale and encouraging preclinical outcomes of responsive, functionalized nanocarriers underscore their potential to advance precision medicine in gout management.

See also the graphical abstract(Fig. 1).

## 1. Introduction

Gout is the most common form of inflammatory arthritis, characterized by episodes of intense joint pain, redness, and swelling. This condition involves crystal-associated arthropathy caused by the accumulation of monosodium urate in the synovial joint. It is linked to hyperuricemia resulting from changes in purine metabolic pathways. Gout is a metabolic disorder marked by recurrent inflammatory arthritis and has a high global prevalence along with significant functional disability (Keenan, 2017[49]). The prevalence of gout is higher in men and increases with age (Jiang et al., 2023[44]). Without proper management, gout can cause chronic joint damage and recurrent flares and has been associated with comorbidities such as cardiovascular and renal complications (Dehlin et al., 2020[26]; Terkeltaub, 2010[106]; Zhang et al., 2024[125]). Factors like genetic predisposition, environmental influences, and unhealthy lifestyle choices, including high-purine diets and excessive alcohol consumption, can contribute to the development of gout (Mikuls, 2022[65]).

**Figure 2:**
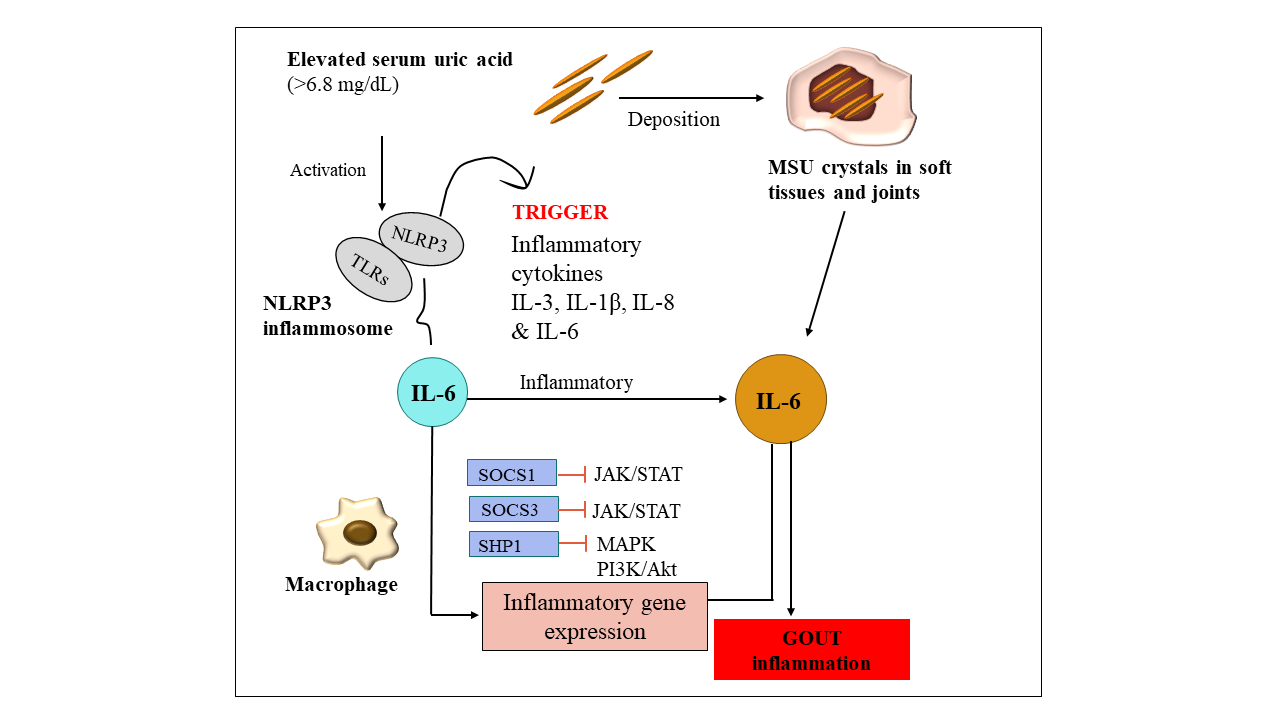
Inflammatory signaling cascade initiated by monosodium urate (MSU) crystal deposition in gout

As depicted in Figure 2(Fig. 2), persistent increase in serum uric acid (sUA) above the solubility threshold (>6.8 mg/dL) encourages the deposition of monosodium urate crystals in soft tissues and joints, which causes inflammation and gradually damages the joints. Reducing sUA levels to less than 6 mg/dL is therefore a primary goal of gout treatment since it aids in the dissolution of preexisting deposits and the inhibition of new crystal formation (Keenan, 2017[49]). Gout inflammation initiates when MSU crystals stimulate the innate immune system via Toll-like receptors (TLRs) and the NOD-, LRR-, and pyrin domain-containing protein 3 (NLRP3) inflammasome, resulting in the secretion of inflammatory cytokines including interleukin-6 (IL-6), interleukin-1 beta (IL-1β), tumor necrosis factor-alpha (TNF-α), and interleukin-8 (IL-8) (Jiang et al., 2023[44]). Cytokines initiate downstream inflammatory signaling pathways, such as the Janus kinase/signal transducer and activator of transcription (JAK/STAT), mitogen-activated protein kinase (MAPK), and phosphatidylinositol 3-kinase/protein kinase B (PI3K/Akt) pathways, resulting in the activation of inflammatory gene expression (Zhang et al., 2024[125]). Interleukin-6 (IL-6) facilitates the recruitment of immune cells and exacerbates local inflammation, thereby aggravating the condition rather than alleviating (Zhang et al., 2024[125]). Elevated IL-6 levels are clinically linked to gouty tophi, joint deformities, and a heightened risk of cardiovascular disease. Under typical circumstances, IL-6 signaling is modulated by proteins including suppressor of cytokine signaling 1 and 3 (SOCS1 and SOCS3) and Src homology region 2 domain-containing phosphatase-1 (SHP1). However, in gout, this regulatory mechanism is frequently surpassed, leading to persistent inflammation.

Several therapeutic strategies have been developed to manage gout based on this pathway. Contemporary anti-hyperuricemic therapies focus on lowering serum urate concentrations by addressing the production, excretion, and degradation of uric acid. Pharmacologic agents commonly utilized comprise xanthine oxidase (XO) inhibitors, including allopurinol (first-line) and febuxostat; uricosurics such as benzbromarone, probenecid, sulfinpyrazone, and lesinurad; and recombinant uricase pegloticase (Dehlin et al., 2020[26]; Terkeltaub, 2010[106]). Uricosurics, although effective, elevate the burden on the kidneys and increase the probability of kidney stone formation, rendering them inappropriate for patients with severe chronic kidney disease (CKD) (Dehlin et al., 2020[26]). Nonsteroidal anti-inflammatory drugs (NSAIDs), glucocorticosteroids, and colchicine are commonly employed for the management of acute gout to alleviate pain and inflammation; however, they are associated with considerable adverse effects and potential drug interactions, particularly in elderly patients or individuals with CKD or diabetes (Terkeltaub, 2010[106]). Recent alternatives targeting IL-1β, including canakinumab, anakinra, and rilonacept, demonstrate significant anti-inflammatory effects (Mikuls, 2022[65]). Nonetheless, their application in gout is restricted, as canakinumab has received approval from the European Medicines Agency (EMA) but not yet from the Food and Drug Administration (FDA) (Pascart et al., 2019[75]; So and Martinon, 2017[101]).

There are many different medications available, however current monotherapies often do not work well to control both the inflammatory cascade and hyperuricemia in gout at the same time. Also, systemic adverse effects, poor absorption, and non-specific distribution make standard therapy even less successful. Nanocarrier-based drug delivery systems have been a viable method in precision medicine in the last several years. These platforms have many benefits, including as better solubility, better pharmacokinetic profiles, targeting specific tissues, and less systemic toxicity. Notably, using nanocarriers to transport a single drug has made a lot of progress in improving treatment outcomes by safeguarding unstable pharmaceuticals, extending circulation time, and making sure that the drug is released in a controlled way at sites of inflammation or disease.

**Figure 3:**
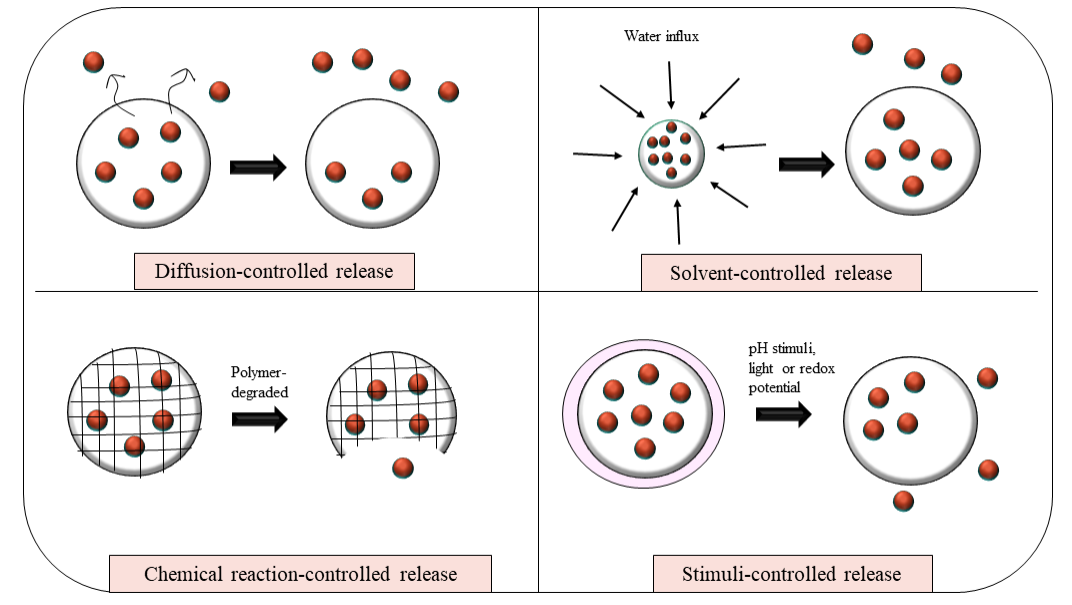
Representative drug release mechanisms from nanocarriers: diffusion-controlled, solvent-controlled, chemical reaction-controlled and stimuli-responsive release

These therapeutic improvements are largely attributed to the sophisticated mechanisms by which nanocarriers regulate the release of encapsulated drugs as shown in Figure 3(Fig. 3). Nanocarriers facilitate controlled drug release through various mechanisms, such as diffusion, solvent-mediated transport, chemical degradation, and stimulus-responsive systems. In diffusion-driven release, the drug moves from the interior of the carrier to the external environment based on concentration gradients, either through a surrounding membrane or within a porous matrix. Solvent-controlled release typically involves water absorption, which induces swelling or osmotic pressure, thereby promoting drug release. Chemical reaction-based mechanisms rely on the degradation of carrier materials or the cleavage of drug-polymer linkages, often triggered by hydrolysis or enzymatic activity. Stimuli-responsive systems, on the other hand, are designed to release their payload in response to specific environmental cues such as pH, temperature, or redox conditions. The kinetics of drug release are largely governed by the physicochemical properties of the drug and its interactions within the carrier matrix. Co-encapsulation strategies, particularly those involving sequential drug loading, enhance stability and modulate release profiles through intermolecular forces like hydrophobic interactions. These complex release behaviors are often described using mathematical models such as the Korsmeyer-Peppas equation, which frequently indicate that diffusion is the primary mechanism controlling drug release.

Researchers are now working on dual-drug encapsulation systems, which will allow two different drugs to be delivered at the same time or one after the other in a single nanocarrier. This is a step ahead from the success of the first system. This method works especially effectively for complicated illnesses like gout, where both inflammation and uric acid buildup need to be treated at the same time. Dual-drug systems deliver synchronised therapeutic benefits, lower the need for repeated doses, and make it easier for patients to follow their treatment by combining an anti-inflammatory and a urate-lowering agent in the same capsule. These systems can also maximize drug effectiveness while minimizing side effects by customising release kinetics and using targeted delivery. This makes them a very appealing option for current gout treatment.

## 2. Current Therapeutic Landscape

### 2.1 Monotherapies

#### 2.1.1 Uric acid-lowering drugs

##### a) Allopurinol

As a first-line urate-lowering treatment (ULT) and XO inhibitor, allopurinol is frequently recommended to treat gout and hyperuricemia (Khanna et al., 2012[50]). Instead of treating renal underexcretion, it lowers the synthesis of uric acid. Allopurinol is still frequently used in a variety of clinical situations and has long been regarded as the standard ULT. It is advised to start with 100 mg daily and gradually increase the dosage by the patient's response and serum uric acid levels (Khanna et al., 2012[50]). Participants in clinical trials like the STOP Gout Trial were started on 100 mg of allopurinol per day and had their dosages titrated up to a median of 400 mg per day (Becker et al., 2005[8]; Helget et al., 2024[37]; O'Dell et al., 2022[70]). 800 mg per day is the highest dosage that is permitted. The main treatment objective in the management of gout is to achieve target blood urate levels below 6 mg/dL, and these dose regimens are in line with the 2016 EULAR recommendations (Richette et al., 2017[88]).

It has also been used to treat acute gout episodes in conjunction with anti-inflammatory medications; however, because of its limited immediate effectiveness, it is typically not recommended during acute flare-ups (Dalbeth et al., 2017[21]; Jarjour et al., 2015[42]; Ke et al., 2017[48]). A purine analogue, allopurinol, differs from another XOD inhibitor, febuxostat, in both structure and metabolism. Febuxostat may be more beneficial for some patient groups, according to comparative trials like the STOP Gout Trial, which has led to a continuous assessment of their cardiovascular safety profiles (Helget et al., 2024[37]). Currently, a specialized study is comparing the cardiovascular outcomes of these two medicines.

Allopurinol's therapeutic value is increased by its indications for the prevention of tumor lysis syndrome and post-chemotherapy hyperuricemia in addition to gout (Yang et al., 2025[122]). It has also been used in conjunction with traditional herbal treatment in some places, like Vietnam (Bardin et al., 2025[7]). All things considered, when administered carefully and under close supervision, allopurinol remains a vital component of long-term gout treatment, providing a tried-and-true method of lowering uric acid levels and enhancing patient results.

##### b) Febuxostat

In the 2000s, febuxostat emerged for clinical application as a non-purine XO inhibitor (Becker et al., 2005[9]; Okamoto et al., 2003[73]). This medication functions to lower uric acid levels in patients with gout by inhibiting xanthine oxidase, effectively reducing sUA levels (Kamatani et al., 2011[45]). Febuxostat differs from allopurinol as it is not a purine analogue and is specifically recommended for patients who cannot tolerate allopurinol or for whom it is contraindicated (Das et al., 1987[23]).

The medication is recommended for those with CKD and does not require adjustments in dosage for mild to moderate liver or kidney impairment. Febuxostat is not advised for individuals with asymptomatic hyperuricemia or notable hepatic impairment (Yang et al., 2025[122]). The safety profile has proven to be reliable, with minimal drug interactions and no significant side effects observed during the 12-week monitoring period. However, it is associated with a slight increase in cardiovascular events and has shown positive cardiovascular outcomes in patients with high uric acid levels (Yang et al., 2025[122]).

Febuxostat is considered a preferred urate-lowering treatment for Chinese patients due to its effectiveness and safety profile. In Japan, it is sold under the name Feburic, with a typical starting dosage of 40 mg daily (Yamanaka et al., 2018[121]). The specific dosage can be found in China, available for ¥12 per pill (Keenan, 2017[49]; Yamanaka et al., 2018[121]). It is recommended to gradually increase the dosage to minimize the chances of gout flare-ups, especially when starting treatment. Febuxostat shows improved effectiveness in lowering urate compared to allopurinol and significantly reduces serum urate levels, even during acute gout attacks. The main goal of febuxostat therapy is to reduce serum uric acid levels to under 6 mg/dL (Keenan, 2017[49]). The clinical effectiveness, safety profile, and ease of administration make it a suitable choice for the long-term treatment of gout and hyperuricemia in diverse patient populations.

#### 2.1.2 Anti-inflammatories

##### a) Colchicine

Colchicine is an anti-inflammatory drug that has been studied for its impact on cardiovascular outcomes in patients with coronary artery disease and gout (Martinon et al., 2006[63]). It obstructs the migration of neutrophils and the NLRP3 inflammasome, consequently affecting cholesterol crystals seen in atherosclerotic arteries (Martinon et al., 2006[63]). Colchicine, despite its anti-inflammatory qualities, has shown variable impact on cardiovascular outcomes. Nevertheless, the medicine is correlated with diminished all-cause mortality in patients undergoing colchicine therapy, and its administration has been associated with a lowered risk of acute coronary syndrome in individuals with confirmed coronary artery disease (Martínez et al., 2015[61]).

Colchicine, a natural chemical extracted from the autumn crocus, has been utilized for millennia in the management of gouty arthritis (Roberts et al., 1987[89]). It is recognized as a first-line for addressing acute gout flares and for flare prophylaxis (Dalbeth et al., 2014[22]). Colchicine influences multiple pro-inflammatory and anti-inflammatory mechanisms associated with gouty arthritis (Dalbeth et al., 2014[22]). It also obstructs microtubule assembly, altering many inflammatory pathways (Ding et al., 1990[27]; Misawa et al., 2013[67]). The therapeutic effects are intricate, including multiple pathways associated with inflammation, indicating possible applicability in other chronic inflammatory diseases.

The prophylactic administration of colchicine is generally characterized by a dosage of 0.5 g taken once or twice day for a minimum duration of one month (Lu et al., 2022[59]). Beyond its recognized function in gout management, colchicine administration has been linked to a reduced probability of gout flare-ups post-vaccination. Research indicates that colchicine may mitigate the likelihood of gout exacerbations following COVID-19 vaccination (Lu et al., 2022[59]).

##### b) Nonsteroidal anti-inflammatory drugs (NSAIDs)

NSAIDs are commonly employed in the management of gout and may be used as prophylaxis when initiating urate-lowering therapy (ULT) to reduce the risk of acute gout flares (Khanna et al., 2012[50]). The American College of Rheumatology (ACR) recommends an integrated approach that includes both pharmacologic and nonpharmacologic strategies, with NSAIDs recognized as a key component in controlling gout symptoms. NSAIDs are commonly prescribed early in gout treatment to manage disease flares and improve clinical outcomes (Oderda et al., 2014[71]).

In clinical studies, attending physicians prescribed NSAIDs at baseline, and patients were instructed to document their use for gout-related symptoms. Gout flares were operationally defined as episodes requiring NSAID use, and the study closely monitored the incidence of these flares that necessitated NSAID treatment.

According to ACR guidelines, NSAIDs are considered first-line agents for acute gout flares, particularly when administered within 24 hours of symptom onset (Khanna et al., 2012[51]). They are often used in combination with colchicine or corticosteroids to provide symptom relief and the European Alliance of Associations for Rheumatology (EULAR) similarly recommends their use during the initial phase of ULT initiation (Dalbeth et al., 2014[22]). Although NSAIDs are typically effective as monotherapy, co-administration with oral glucocorticoids is generally avoided due to the increased risk of gastrointestinal bleeding (Abhishek and Cipolletta, 2025[1]). For patients in whom NSAIDs, colchicine, or corticosteroids are contraindicated, interleukin-1 (IL-1) inhibitors such as anakinra may serve as an alternative (Shaukat et al., 2020[96]).

##### c) Glucocorticoids

Glucocorticoids represent a category of steroid hormones frequently employed in managing a range of inflammatory disorders, such as gout. In the context of gout management, they are typically employed to alleviate inflammation and discomfort during acute flare-ups. Nonetheless, caution is advised regarding their use in patients experiencing decompensated heart failure, as it may lead to adverse cardiovascular outcomes (Shaukat et al., 2020[96]).

Glucocorticoids may be delivered via oral administration or through intra-articular injections, especially in cases where one or two joints are involved and appropriate for direct injection. This approach is frequently selected even in the absence of randomized controlled trial (RCT) evidence directly validating the effectiveness of glucocorticoids in these situations (Abhishek and Cipolletta, 2025[1]). The standard dosage of glucocorticoids is contingent upon the specific joint affected. For example, a dosage of 10 mg is typically sufficient for interphalangeal joints, while larger joints like the knees, ankles, and wrists might necessitate doses reaching up to 40 mg (Abhishek and Cipolletta, 2025[1]). In these instances, long-acting glucocorticoids such as methylprednisolone or triamcinolone are typically favoured due to their prolonged anti-inflammatory effects.

Prior to the prescription of glucocorticoids, it is essential to exclude septic arthritis in order to reduce the risk of administering steroid therapy to an infected joint. This diagnostic step, while essential, has the potential to postpone the start of treatment. In specific clinical scenarios, glucocorticoids can be utilized when patients cannot tolerate alternative medications or when those medications are deemed contraindicated (Khanna et al., 2012[51]). These provide an alternative approach for preventing flares in gout patients who do not achieve sufficient results with conventional treatments like XO inhibitors or uricosuric agents.

Low-dose glucocorticoids, including prednisolone, are considered second-line prophylactic anti-inflammatory therapies for managing gout flares (Keenan, 2017[49]). Their application may be essential for individuals who suffer from recurrent flares and are unable to utilize alternative anti-inflammatory treatments due to existing health issues or contraindications. The main objective of treating gout, which involves the administration of glucocorticoids, is to lower sUA levels to under 6 mg/dL and to manage inflammation efficiently.

### 2.2 Adverse effects of existing drugs

Current monotherapies are still the main way to treat gout, but their side effects show that they have serious clinical limitations as shown in Table 1(Tab. 1) (References in Table 1: Bardin et al., 2025[7]; Cipolletta et al., 2025[18]; Da Silva et al., 2006[20]; Dalbeth et al., 2014[22]; Eleftheriadis et al., 2017[28]; Hande et al., 1984[35]; Helget et al., 2024[37]; Huscher et al., 2009[41]; Martingano et al., 2025[62]; Office of the Surgeon General (US), 2004[72]; Piper et al., 1991[80]; Roddy et al., 2023[90]; Sandler et al., 1991[91]; Sattui and Gaffo, 2016[92]; Schakman et al., 2008[94]; Singh and Cleveland, 2019[98]; Stamp, 2014[103]; Tateishi et al., 1997[105]; Terkeltaub et al., 2011[107]; Wei et al., 2004[116]), especially in those who have other health problems at the same time. Drugs that decrease uric acid, like allopurinol and febuxostat, can help with hyperuricemia, but they also raise major safety concerns, including hypersensitivity syndromes, liver problems, and heart problems. Allopurinol is especially dangerous for people with kidney problems since it can build up oxypurinol, which can make it more poisonous. Febuxostat is frequently the best choice in these situations, but it can still cause problems including atrial fibrillation and liver problems. On the other hand, anti-inflammatory drugs, which are often used to treat acute flares, raise a new set of issues. People often have stomach problems, kidney problems, and a higher risk of heart disease when they take NSAIDs. Colchicine works well to target neutrophil-driven inflammation, but it has a narrow therapeutic range and can cause neuromuscular toxicity and life-threatening systemic effects if taken in too high a dose. Glucocorticoids are widely used to quickly treat symptoms, but they also increase the risk of long-term problems such osteoporosis, myopathy, and unstable cardiovascular health. These bad outcomes show how hard it is to find the right balance between therapeutic efficacy and systemic burden in gout pharmacotherapy. They also show why treatment must be personalized to each patient's individual weaknesses.

### 2.3 Evaluating the compatibility of uric acid-lowering and anti-inflammatory agents

Considering these unfavorable profiles, especially in those with renal or cardiovascular sensitivities, it is crucial to gain a more profound insight into the interactions between frequently co-administered medications and urate-lowering therapies to enhance treatment safety and efficacy. Allopurinol and febuxostat are both effective at inhibiting xanthine oxidase, yet they exhibit notable differences in their elimination pathways. Allopurinol relies mainly on renal clearance, while febuxostat is eliminated through both hepatic and renal processes. The variations observed impact their vulnerability to pharmacokinetic interactions. Medications that negatively affect kidney performance or disrupt renal transport mechanisms can markedly decrease the clearance of allopurinol and increase the buildup of oxipurinol, thereby raising the potential for toxicity. Febuxostat, although not significantly impacted by renal impairment, may still raise issues due to its inhibition of certain cytochrome P450 enzymes and efflux transporters such as BCRP. The following section provides a comparative overview of the pharmacokinetics of these urate-lowering agents in relation to commonly prescribed anti-inflammatories, detailing aspects such as absorption, distribution, metabolism, elimination, and the potential for drug-drug interactions. This knowledge is crucial for developing innovative strategies to combine medications and exploring potential options for co-encapsulation in advanced gout treatments.

The comparative pharmacokinetic Table 2(Tab. 2) (References in Table 2: Ahmad et al., 2002[3]; Bekker et al., 2018[10]; Ben-Chetrit et al., 1994[11]; Brutzkus et al., 2025[12]; Bushra and Aslam, 2010[13]; Buvanendran and Reuben, 2008[14]; Chappey and Scherrmann, 1995[16]; Davies and Anderson, 1997[24][25]; Ferry et al., 1988[29]; Finkelstein et al., 2010[30]; Gong et al., 2012[32]; Pickup, 1979[79]; Rainsford, 2009[84]; Rao and Knaus, 2008[85]; Reiter et al., 1983[87]; Todd and Clissold, 1990[109]; Todd and Sorkin, 1988[110]; Türck et al., 1996[111]; Weiner, 2018[117]) indicates how various drugs that are regularly administered with allopurinol can modify its pharmacokinetics and safety, especially through renal and transporter-mediated pathways. People know that allopurinol is easy to absorb via oral route (about 90 %), that it reaches peak plasma levels fast (1.5 hours for allopurinol and 4.5 hours for its active metabolite, oxipurinol), and that the kidneys primarily get rid of it (about 80 % through the urine). It is not a P-glycoprotein (Pgp) substrate and does not go through cytochrome P450 (CYP450) metabolism, which means it is considerably less likely to have direct drug interactions that affect metabolism. But since it relies on the kidneys to get rid of it, any treatment that changes how the kidneys work or how they move things around, especially organic anion transporter 3 (OAT3), can modify how quickly it leaves the body. This is particularly important for NSAIDs, which are typically used alongside allopurinol to help individuals with gout feel better and reduce swelling.

NSAIDs such as ibuprofen, diclofenac, naproxen, meloxicam, celecoxib, and etoricoxib are not Pgp substrates, but they all have one important pharmacologic property in common: they can damage kidney function. Even though they all get rid of waste and break down food in distinct ways, many of them either directly impede renal transporters or limit glomerular filtration by blocking prostaglandins. Urine breaks down and gets rid of ibuprofen, for example. It has been shown to stop OAT3, a transporter that helps the kidneys get rid of medicines. When ibuprofen blocks OAT3, it makes it harder for the kidneys to get rid of allopurinol or oxipurinol, which might boost their levels in the blood. This makes the chance of poisoning higher. Diclofenac also substantially inhibits OAT3 and is broken down by cytochrome P (CYP) enzymes (CYP1A2, 2C19, 2C9, and 2D6). However, this CYP activity does not relevant for allopurinol because it does not employ this metabolic route. It is still a big concern for drug interactions since it changes how pharmaceuticals are moved around in the kidneys. Naproxen does not stop CYP450, but it can affect the kidneys, which could make it harder for the body to get rid of allopurinol. Like allopurinol, meloxicam has a long half-life, and the liver breaks it down the most. It is also known to disrupt CYP2C9 and CYP3A4, but more importantly, it may hurt how the kidneys work and make it difficult for them to get rid of allopurinol.

Celecoxib and etoricoxib are both selective cyclooxygenase-2 (COX-2) inhibitors that are extensively used and broken down in the liver by a number of CYP enzymes. CYP1A2, 2C9, and 2D6 break down celecoxib, while etoricoxib stops CYP1A2, 2C19, 2C9, and 3A4 from working and is a substrate of CYP2D6 and 3A4. Even while they do not directly change how allopurinol is broken down, both may nonetheless make the kidneys operate less properly. This lower renal clearance may also slow down the elimination of allopurinol, which might cause both the parent drug and its metabolite, oxipurinol, to build up. This effect may be stronger if you take meloxicam for a long time (20 to 42 hours) or etoricoxib for a long time (22 hours).

Colchicine is not nephrotoxic, although it may interact with allopurinol in a different way. It is a Pgp substrate that distributes all over the body and is eliminated by the kidneys and bile. CYP450 pathways do not break down colchicine very effectively (it blocks CYP2D6 and CYP3A4 and is a substrate of CYP3A4), but how quickly it leaves the body depends on how well the kidneys perform. If the kidneys do not work right or if colchicine is used with other drugs that use the same elimination pathways in the kidneys, such allopurinol, it can build up. This can make the medicine more harmful, leading to myopathy and stomach difficulties, especially in patients with kidney problems or when colchicine is administered more than once. Allopurinol and colchicine do not directly impact each other through hepatic metabolism, but they both depend on renal pathways, which is a clinically important interaction issue, especially when the kidneys are not performing well. When it comes to how it might interact with allopurinol, prednisolone looks to be the safest of the drugs on the list. It is a Pgp substrate and is quite bioavailable, however how it is broken down depends on the dose and largely happens through non-CYP hepatic pathways. It is largely eliminated from the body through urine (>98 %) and does not stop or start CYP enzymes or interfere with renal drug transporters such OAT3. Also, prednisolone does not seem to hurt the kidneys and has a short half-life. Even in persons with mild to moderate kidney problems, prednisolone generally will not modify how allopurinol works in the body or make it more hazardous.

In conclusion, allopurinol has a minimal impact on liver enzymes, primarily because it does not affect the Pgp and CYP450 enzymes. But it is highly susceptible to changes in pharmacokinetics when taken with medications that influence renal function or share renal transport systems because it relies on renal excretion. NSAIDs such diclofenac, ibuprofen, naproxen, meloxicam, celecoxib, and etoricoxib can limit the amount of allopurinol that the kidneys can pass by either inhibiting OAT3 or lowering blood flow to the kidneys. Colchicine acts differently in the body, but it's still a concern because it competes with the kidneys to get rid of it. Prednisolone is still an exception because it does not interact with many other drugs. These results highlight how crucial it is to keep an eye on how well your kidneys are working and to be careful about adjusting the dose when you take these drugs with allopurinol, especially if you already have kidney problems or have been taking the drugs for a long time.

On the other hand, Table 3(Tab. 3) (References in Table 3: Ahmad et al., 2002[3]; Bekker et al., 2018[10]; Ben-Chetrit et al., 1994[11]; Brutzkus et al., 2025[12]; Bushra and Aslam, 2010[13]; Buvanendran and Reuben, 2008[14]; Chappey and Scherrmann, 1995[16]; Davies and Anderson, 1997[24][25]; Ferry et al., 1988[29]; Finkelstein et al., 2010[30]; Gong et al., 2012[32]; Grabowski et al., 2011[33]; Hu and Tomlinson, 2008[39]; Pickup, 1979[79]; Rainsford, 2009[84]; Rao and Knaus, 2008[85]; Todd and Clissold, 1990[109]; Todd and Sorkin, 1988[110]; Türck et al., 1996[111]; Weiner, 2018[117]) shows how febuxostat works and how it might interact with other drugs that are widely used to treat gout and other inflammatory conditions. Febuxostat has a high oral bioavailability, which means that about 85 % of the medicine is absorbed and peak plasma concentrations are attained within 1 to 1.5 hours. It spreads out evenly throughout the body, with a steady-state volume of distribution between 29 and 75 liters. Febuxostat is broken down and removed by both the liver and the kidneys. Almost half of the amount that is given is found in urine, mostly as metabolites. It is also known to stop cytochrome P450 enzymes including CYP1A2, CYP2C19, and CYP2C9 from working. Febuxostat also stops the breast cancer resistance protein (BCRP), which is an important drug efflux transporter that helps the body absorb drugs and spread them throughout the body. The medications in the table, such as ibuprofen, naproxen, meloxicam, diclofenac, etoricoxib, prednisolone, and colchicine, do not have any major pharmacokinetic interactions with febuxostat. Many of these medications are broken down by liver enzymes or removed by the kidneys, but they do not seem to use the same transporters or enzyme routes that febuxostat does. Ibuprofen and naproxen, for example, are broken down a lot and mostly leave the body in urine, but they do not have anything to do with BCRP. Meloxicam and diclofenac affect different CYP enzymes, but they do not have any clinically significant overlap with febuxostat's inhibitory profile. The kidneys mostly get rid of prednisolone, and it does not change the cytochrome enzymes or transporters that febuxostat does. Colchicine needs CYP3A4 and P-glycoprotein to break down and move around, but the routes that were looked at did not show any interaction with febuxostat. Celecoxib is the sole medicine in the table that might cause an interaction. It is a recognized substrate of BCRP, and febuxostat's blocking of this transporter could make celecoxib more easily absorbed and spread throughout the body. Depending on the patient's other health problems, dose, and metabolic status, this interaction could have different impacts on their health. However, the possibility of higher celecoxib levels could make the risk of side effects higher, thus it should be taken into account when giving the two drugs together.

In conclusion, febuxostat interacts well with most of the drugs that are typically administered together to treat gout. The data show that there is a very low risk of metabolic or transporter-mediated interactions, except for celecoxib, where febuxostat may enhance drug exposure by blocking BCRP. When looking at possible drug-drug interactions in patients taking febuxostat as part of combination therapy, this shows how important it is to look at both enzymatic and transporter-mediated pathways.

## 3. Nanocarrier In Drug Delivery

A nanocarrier is a nanoscale delivery system developed to transport therapeutic agents, including drugs, DNA, or nucleic acids, to targeted cells or tissues within the body (Rahmani et al., 2025[83]; Sawant et al., 2006[93]). These systems optimize the effectiveness of drug delivery by enhancing the stability, solubility, and bioavailability of therapeutic agents, all while reducing side effects. They demonstrate notable efficacy in traversing biological barriers, including the blood-brain barrier, thereby enabling precise and regulated drug delivery at the intended site of action (Wohlfart et al., 2011[120]).

Nanocarriers can be designed to react to particular stimuli, including variations in pH or temperature, facilitating targeted release in pathological conditions such as tumors or inflamed tissues. These can be engineered to focus on particular cells or tissues, minimizing harm to healthy cells (Sawant et al., 2006[120]). These carriers are generally colloidal nanoparticles, with dimensions spanning from 1 to 100 nanometres, and therapeutic applications frequently necessitating sizes under 200 nm (Peer et al., 2007[76]; Singh and Lillard, 2009[100]). Their diminutive dimensions and extensive surface area enhance drug delivery and bolster stability in comparison to traditional dosage forms. They provide excellent biocompatibility, prolonged circulation, and consistent drug release over time (Chamundeeswari et al., 2019[15]).

**Figure 4:**
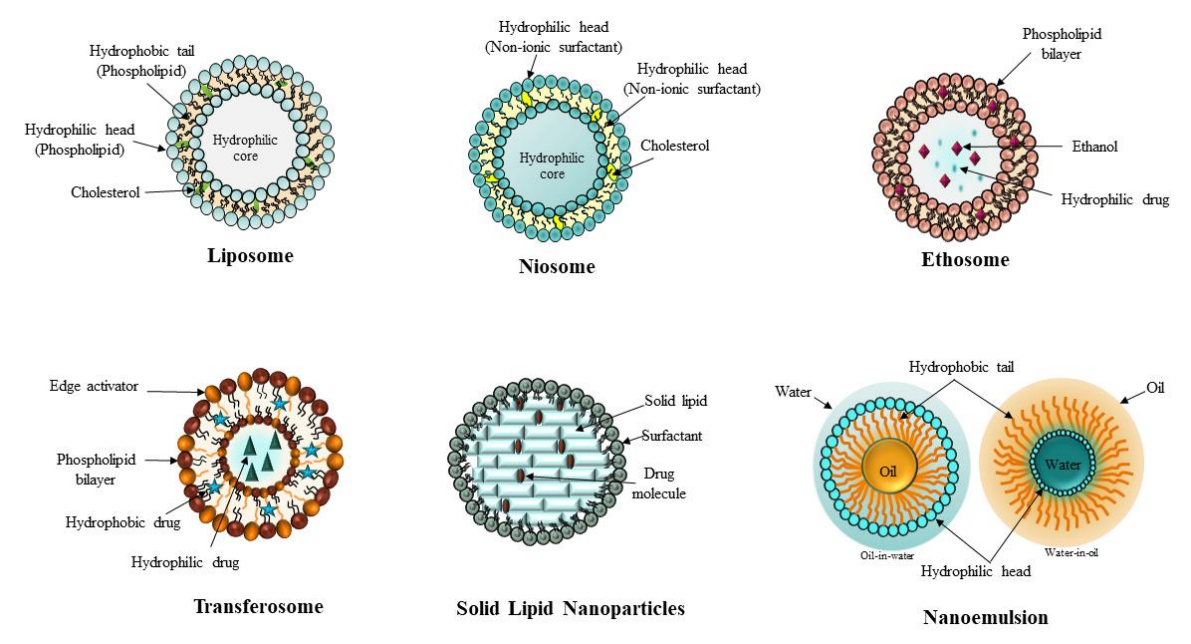
Structure of various nanocarriers

Nanocarriers can be made from a range of materials, such as lipids, polymers, and inorganic substances, providing opportunities for versatile design in various shapes and sizes (Chou et al., 2011[17]; Zhang et al., 2021[123]). Common types of nanocarriers encompass liposomes, micelles, dendrimers, solid lipid nanoparticles, polymeric nanoparticles, and additional variants (Figure 4(Fig. 4)). They serve an essential function in nanobiotechnology, facilitating effective drug delivery and diagnostics. The creation and analysis of nanocarriers encompass a range of methods aimed at confirming their safety and efficacy for use in clinical settings. Lipid-based nanoparticles have been utilized to encapsulate microbial nucleic acids, aiding in their delivery and improving immune responses (Rahmani et al., 2025[83]).

To further understand their potential in drug delivery, it is essential to examine the different types of nanocarriers, highlighting their structural characteristics, composition, and specific advantages that contribute to improved therapeutic efficacy.

### 3.1 Types of nanocarriers

#### 3.1.1 Liposomes

A liposome is a spherical vesicle composed of lipid bilayers capable of encapsulating both hydrophilic and lipophilic drugs, making it a versatile drug delivery system, as illustrated in Figure 4(Fig. 4). Liposomes are colloidal vesicles with a bilayer structure that encapsulate aqueous volumes, made from either natural or synthetic phospholipids (Atre and Rizvi, 2024[6]). Their size can range from 0.02 to 10 μm, and they can be designed for targeted drug delivery purposes (Shukla et al., 2018[97]). The bilayer structure enables the encapsulation of both hydrophilic drugs within the aqueous core and hydrophobic drugs in the bilayer membrane, thereby enhancing therapeutic efficacy (Peng et al., 2023[77]). The architecture of liposomes facilitates significant drug loading efficiency, which is essential for achieving effective treatment results.

Liposomes offer numerous benefits compared to conventional drug delivery systems, such as targeted delivery, controlled release, and protecting encapsulated drugs from degradation. Thus, they enhance the solubility and stability of drugs, reduce toxic side effects, and improve the pharmacokinetics and biodistribution of encapsulated substances, thereby enhancing therapeutic efficacy and patient quality of life. The distinctive characteristics encompass biocompatibility, biodegradability, and the capacity to enhance drug solubility and stability.

Liposomes are categorised into different types, such as small unilamellar vesicles and multilamellar vesicles. Stealth liposomes, a specific variant of liposomes, undergo modification with hydrophilic polymers such as polyethylene glycol (PEG) to improve stability and minimize opsonisation, which in turn extends their circulation time within the bloodstream (Senjab et al., 2024[95]). The physical stability of liposomes is affected by factors such as lipid composition, size distribution, and storage conditions, all of which are essential for their clinical application. Different techniques, including thin-film hydration and ethanol injection, are utilized to create liposomes.

#### 3.1.2 Niosomes

One method of drug delivery is via niosomes, which are vesicles made of non-ionic surfactant and are not toxic. These are microscopic vesicles made of non-ionic surfactants that, because of their amphiphilic nature, form closed bilayer structures. Amphiphiles in aqueous solutions self-assemble to generate niosomes, which are vesicles based on nonionic surfactants. Like liposomes, they form closed bilayer structures, as shown in Figure 4(Fig. 4).

Both hydrophilic and hydrophobic medications can be encapsulated by niosomes, increasing the potential applications for these molecules. They improve drug delivery by penetrating the stratum corneum. They can act as drug transporters and encapsulate aqueous solutes. When preparing niosomes for high encapsulation effectiveness, sorbitan monostearate, or Span 60, is frequently utilized. Equimolar combinations of surfactants, such as Tween 80 and Span 80, are used to create niosomes (Machado et al., 2018[60]). The components of niosomes, such as stabilizers and non-ionic surfactants, affect their effectiveness. For an example, cholesterol helps stabilize the structure of niosomes by making the bilayer more hydrophobic and lowering its surface energy. This not only supports the formation of smaller particles but also boosts how well the formulation works (Zolkepli et al., 2025[127]).

Because niosomes can form vesicular carriers, they offer efficient drugs delivery. In a range of biological settings, they demonstrate strong chemical stability. Because of their stability and affordability, niosomes are regarded as a liposome substitute. Compared to liposomes, they have better chemical stability and are less expensive. For medication delivery methods, niosomes are being investigated as liposome substitutes. Besides, food, agricultural, pharmaceutical, and cosmetics sectors all also use niosomes. Applications for niosomes are numerous and include gene and drug delivery.

#### 3.1.3 Ethosomes

Ethosomes are flexible lipid vesicles primarily made up of phospholipids, ethanol, and water. Their design aims to improve transdermal drug delivery by enabling deeper skin penetration and addressing the challenges posed by the epidermal barrier, with their structural composition illustrated in Figure 4(Fig. 4). In contrast to traditional systems, ethosomes demonstrate enhanced efficacy in the delivery of both hydrophilic and lipophilic drugs, particularly those characterized by limited skin permeation and low oral bioavailability. The high ethanol content of ethosomes, usually between 20 % and 45 %, is a significant characteristic that enhances membrane fluidity and improves drug permeability (Akhtar et al., 2022[4]). The inclusion of ethanol, paired with edge activators like Tween 20, enhances their effectiveness in facilitating drug transport across biological membranes, enabling the concentration of drugs in deeper layers of the skin.

Ethosomal formulations are straightforward, secure, and efficient, providing controlled drug release as evidenced by zero-order release in in-vitro studies. Sonicated ethosomes demonstrate favorable attributes, including reduced vesicle size and improved entrapment efficiency, both of which are essential for optimal skin permeation and increased therapeutic effectiveness. Ethosomes have demonstrated successful applications across multiple domains, such as photodynamic therapy, cosmeceuticals, and other topical drug formulations, surpassing liposomes and hydroethanolic solutions in penetration depth and drug delivery efficiency.

#### 3.1.4 Transferosomes

Figure 4(Fig. 4) illustrates transferosomes, which represent extremely flexible and malleable vesicular systems composed of phospholipids and an edge activator. The efficiency of drug delivery is improved by their ability to readily deform and penetrate biological barriers, such as the skin and mucosal membranes, due to their distinctive structure. The presence of edge activators enhances bilayer fluidity, thereby contributing to their remarkable elasticity and deformability. 

These vesicles possess the ability to encapsulate hydrophilic, lipophilic, and amphiphilic drugs, thereby serving as effective carriers for a diverse array of active compounds. Transferosomes demonstrate markedly enhanced skin penetration, superior drug bioavailability, and extended drug release while maintaining low toxicity in comparison to traditional liposomes and ethosomes (Rahman et al., 2020[82]). They are also straightforward to expand without relying on additives that do not meet pharmaceutical standards.

Transferosomes have been effectively utilized in numerous drug delivery investigations, such as transdermal insulin delivery and improved voriconazole formulations for treating fungal infections (Marwah et al., 2016[64]). The addition of components, such as clove oil, has demonstrated potential in mitigating side effects like nephrotoxicity while simultaneously improving therapeutic efficacy. Their enhanced permeability and capacity to circumvent first-pass metabolism render them a highly promising system for both topical and transbuccal drug delivery.

#### 3.1.5 Solid Lipid Nanoparticles

Solid lipid nanoparticles (SLNs) are lipid-based drug delivery systems that have been developed since the 1990s, offering enhanced stability and controlled drug release (Marwah et al., 2016[64]). Their structural architecture is depicted in Figure 4(Fig. 4). They consist of solid lipids, surfactants, and water. Their solid structure, which is characterized by diameters that typically range from 50 to 1000 nm, sets them apart from hollow vesicular carriers (Miller and Spence, 1998[66]). SLNs are composed of physiological lipids, demonstrating an absence of biotoxicity and a notable reduction in toxicity effects.

These nanoparticles significantly improve drug bioavailability by optimizing absorption mechanisms, including intestinal uptake and lymphatic transport. These systems are capable of encapsulating both hydrophilic and hydrophobic, facilitating precise delivery, prolonged release, and safeguarding against degradation in the gastrointestinal tract. SLNs demonstrate promise in augmenting corneal absorption, enhancing ocular bioavailability, and facilitating autoclave sterilization for ocular formulations.

Methods for preparing SLNs encompass both hot and cold homogenization as well as microemulsions, rendering them appropriate for large-scale production (Gasco et al., 2009[31]). While SLNs offer certain benefits, they also face challenges such as limited drug loading capacity and elevated water content in dispersions. These issues have been tackled by the advancement of nanostructured lipid carriers (NLCs), which exhibit enhanced drug loading and release characteristics. SLNs have shown promising results in drug delivery across a range of applications, such as transdermal, ocular, and targeting the central nervous system. In the treatment of diabetes, research has focused on improving the pharmacokinetics of medications such as empagliflozin, suggesting their potential to enhance therapeutic results.

#### 3.1.6 Nanoemulsion

Characterized by droplet sizes typically ranging from 10 to 1000 nm, nanoemulsions are fine oil-in-water (O/W) or water-in-oil (W/O) dispersions stabilized by surfactants. A visual representation of their internal structure and composition is captured in Figure 4(Fig. 4). These transparent or translucent emulsified systems enhance the bioavailability of poorly soluble drugs and herbal products, making them suitable carriers for drug delivery systems. The small droplet size provides increased surface area, stability, and an occlusive effect, improving drug release profiles and dermal drug permeation.

Nanoemulsions are composed of oils, surfactants, and an aqueous phase. They protect active ingredients from oxidation, hydrolysis, and volatilization, contributing to their effectiveness in both oral and topical drug delivery. Their ability to enhance skin penetration and improve the therapeutic efficacy of active compounds makes them valuable for dermatological and cosmetic applications.

Several studies have explored nanoemulsion formulations for different purposes, such as Phyllanthus emblica branch extract for potential skin applications, AG-loaded nanoemulsions to improve oral bioavailability and anti-inflammatory effects, and Rapanea ferruginea extract for enhanced topical delivery and anti-inflammatory activity. High entrapment efficiency, stability, and nanoscale droplet sizes have been observed in these formulations, confirming their effectiveness as drug delivery carriers.

### 3.2 Nanocarriers in the treatment of gout

A variety of nanocarriers, including liposomes, niosomes, and ethosomes, are employed in the treatment of gout. These carriers are designed to specifically gather in inflamed joints and provide therapeutic agents right at the site of action. The aim of these nanocarrier systems is to boost drug efficacy, reduce side effects, and enhance therapeutic outcomes in gout management. Expanding upon this, the use of these nanocarriers in gout treatment has been explored, emphasizing the encapsulation of individual therapeutic agents to improve their effectiveness and address the challenges linked to traditional formulations.

An example of this is allopurinol, which has been encapsulated in niosomes as well as in chitosan-based nanoparticles (Kandav et al., 2019[46]; Singh et al., 2017[99]). The formulations lead to a notable decrease in uric acid levels and inflammation, offering sustained drug release, enhanced solubility in biological fluids, improved bioavailability, and lower toxicity in comparison to free allopurinol (Qi et al., 2024[81]). The oral bioavailability of febuxostat has been greatly improved through its incorporation into nanoemulsions and a self-nanoemulsifying drug delivery system (Heena et al., 2022[36]). The formulations enhance permeation, especially for transdermal delivery, owing to their small droplet size and low viscosity, while also minimizing drug loss prior to reaching the target site (Al-Amodi et al., 2020[5]).

Colchicine has been incorporated using nanoemulsions and nanoparticles derived from chitosan (Aboumanei and Fayez, 2021[2]; Parashar et al., 2022[74]). The nanocarriers enhance the retention of the drug within the joint cavity for intra-articular injections, demonstrate improved antigout activity relative to solution forms, and facilitate sustained release while avoiding first-pass metabolism during transdermal delivery. Furthermore, myricitrin has been effectively incorporated into proliposomes, showcasing improved bioavailability and diminished liver and kidney toxicity in comparison to the unencapsulated drug (Weng et al., 2019[118]).

Copper oxide nanoparticles have been investigated as a potential therapeutic strategy for gout treatment, demonstrating a notable decrease in uric acid levels and enhanced biocompatibility (Herdiana et al., 2025[38]). In comparison to copper sulfate, copper oxide nanoparticles demonstrate reduced toxicity to the kidneys and liver, positioning them as a more favorable option. Encapsulated in solid lipid nanoparticles, Shogaol has demonstrated improved bioavailability and heightened efficacy against gout. The SLNs exhibited characteristics of being small (under 100 nm), spherical, and smooth, alongside a notable encapsulation efficiency of 87.67 % (Herdiana et al., 2025[38]). The *in vitro* release profiles were notably improved, and they demonstrated enhanced oral bioavailability when compared to free Shogaol (Wang et al., 2018[114]). In addition to these, alpha-phellandrene has been formulated into a transdermal ethosomal gel, which enhances skin deposition and permeation, contributing to effective anti-gout activity (Valsalan Soba et al., 2021[112]).

Furthermore, various non-steroidal anti-inflammatory drugs (NSAIDs) have been incorporated into nanocarrier systems to improve their therapeutic profiles in gout management. Etoricoxib, encapsulated in niosomes, demonstrates improved entrapment efficiency and sustained release, enhancing its anti-inflammatory effects (Ravalika and Sailaja, 2017[86]). Lornoxicam, formulated as a liposomal gel, provides prolonged release and improved topical delivery efficiency, while naproxen, loaded into niosomes, exhibits improved drug loading and controlled release, resulting in enhanced anti-inflammatory activity (Krishna Sailaja and Shreya, 2018[55]; Kumbhar et al., 2013[56])

In addition to these, other nanocarrier systems like zinc oxide nanoparticles, turmeric nanoparticles (T-NPs), and bio-nanoparticles have been recognized for their promising potential in the treatment of gout. Zinc oxide nanoparticles play a significant role in alleviating oxidative stress and managing gout arthritis, whereas turmeric nanoparticles have shown potential as efficient anti-gout agents (Kiyani et al., 2019[52][53]). Research has shown that different types of nanoparticles, such as copper oxide and bio-nanoparticles, significantly decrease ankle inflammation and the biochemical markers linked to gout. The various approaches utilizing nanocarriers highlight the promise of single-drug encapsulation strategies in addressing the challenges associated with free drugs. These strategies offer enhanced drug solubility, improved bioavailability, targeted delivery, and minimized systemic toxicity, thereby significantly improving therapeutic outcomes in the management of gout.

Expanding on the previous section, which highlighted the selection of nanocarriers for specific drug pairings, this section focuses on the functionalization strategies used to tailor these systems for advanced therapeutic performance. Functionalization typically involves modifying the surface or internal structure of nanocarriers to enable features such as sequential drug release, targeted delivery, and responsiveness to physiological stimuli. These modifications are particularly important in dual-drug delivery systems, where precise control over the release profile and biodistribution of each agent is crucial. Nanocarriers such as liposomes, niosomes, micelles, and polymeric nanoparticles are commonly engineered through these approaches to enhance treatment specificity and efficacy in complex diseases.

### 3.3 Functionalization strategies in nanocarrier

A lot of research has been done on how to improve site-specific delivery and cellular absorption by changing the surface with targeting ligands such aptamers, receptor ligands, antibodies, folic acid, and chondrocyte-targeting motifs like the chondrocyte affinity peptide DWRVIIPPRPSA (Hu et al., 2021[40]; Muzzalupo and Mazzotta, 2019[68]; Pi et al., 2011[78]). In the treatment of osteoarthritis, acid-activatable poly-beta-amino-esters (PBAEs) have been chemically modified to enhance their targeting and uptake in cartilage (Kang et al., 2020[47]). The net charge in cationic peptide carriers (CPCs) has also been optimized to help drugs stay in cartilage longer. Dex/FA-Oxi-αCD uses folic acid to target inflammatory joints in people with rheumatoid arthritis (Liu et al., 2020[57]).

Another important strategy is to use polymers for functionalization. For instance, PEGylation makes tumors more likely to gather and nanoparticles more likely to move about, but it might make targeting less effective. This has led to the creation of nanoparticles with sandwich-like surfaces that may stay in the body longer and target specific areas. These nanoparticles have matrix metalloproteinases (MMP)-sensitive linkers that let the PEG layers split, which makes it easier for cancer cells to adhere to the specific ligands (Liu et al., 2024[58]). Researchers have also made surface-engineered nanoparticles that can be programmed to circulate and target tumors utilising enzyme-sensitive mPEG-Pep-PCL and folate-containing FA-PEG-PCL copolymers. These nanoparticles have a sandwich shape with a hydrophobic inner core, a targeted middle layer, and a long-circulating outside layer. This makes it easier for drugs to build up at cancer sites.

Responsive nanocarrier technologies have not yet been used in clinical settings to treat gout. However, their basic design principles, which were first studied in cancer and inflammatory disorders, suggest that they could be very useful for treating gout in the future. These systems use internal or external cues to release drugs at the right time, giving them better pharmacokinetic control and localized administration. You can make carriers like lipidosomes, micelles, and carbon nanotubes with enzyme-labile connections that break down when certain enzymes are present. For example, MMPs, which are commonly overexpressed in tumor microenvironments, have been employed to help in localized release. During gout flares, inflamed synovial tissues may be able to use similar enzymatic signals, and higher protease levels might be used as a targeted release trigger.

pH-responsive and ROS-responsive nanocarriers seem to be the best options for gout since the response of the body to acute flares is so different. During these times, inflammatory cells, metabolic waste, and lactic acid build up in the joints, making the area more acidic. To take advantage of this, pH-sensitive nanocarriers are usually made using chemicals like resveratrol and urocanic acid in emulsion-based ways (Song et al., 2023[102]). These systems stay stable at normal body pH, but they release their encapsulated drugs when they come into contact with acidic environments. This feature is especially useful for targeting joints that are swollen with gout as the pH range at the inflamed joint is slightly acidic.

Vesicular systems, like niosomes or liposomes, that are made using cholesteryl hemisuccinate or hexadecyl-poly(acrylic acid) copolymers have been shown to release drugs and be taken up by cells better at low pH (Gugleva et al., 2024[34]). These traits make them good candidates for getting drugs to places where gout-related inflammation is happening, which is usually acidic. Simultaneously, Boric esters, thioketal linkers, or disulphide bonds have been employed to create ROS-responsive nanocarriers, which are cleaved in oxidative environments (Koo et al., 2008[54]; Tao and He, 2018[104]; Wilson et al., 2010[119]). In gout, the activation of neutrophils and macrophages throughout the inflammatory cascade makes a lot of reactive oxygen species (ROS), which makes oxidative stress worse in the area (Zhang et al., 2024[124]). ROS-responsive carriers can consequently allow the release of urate-lowering or anti-inflammatory medicines in targeted areas of inflamed joints. Many of these systems also have the ability to scavenge ROS on their own, which protects tissues even more by reducing oxidative damage.

Other responsive modalities, such nanocarriers that are triggered by light, have also showed potential in cancer research. Platforms that use bis-(alkylthio) alkene (BATA) and chlorin e6 (Ce6) to make light-induced singlet oxygen (^1O2) for drug release, and azobenzene derivatives that let you regulate space through light-activated prodrug activation (Liu et al., 2020[57]). Localized phototriggered systems may have future potential in inflammatory conditions, although it is yet unclear how they will work in deeply located joint tissues.

Nanocarriers that have been created on the surface have also been shown to increase circulation time and tissue targeting. For instance, albumin-binding domains help inflammatory macrophages (like RAW264.7 cells) take up more of the drug, while pH-sensitive histidine residues and solubility-enhancing guanidinium groups in liposomal structures make it easier for the drug to interact with acidic, inflamed tissues (Liu et al., 2020[57]). These designs have mostly been utilized with medications like paclitaxel and curcumin but modifying them for gout-specific treatments could help combo therapy work better.

With a clearer understanding of how nanocarriers can respond to the inflammatory and oxidative environment characteristic of gout, it becomes essential to investigate how these systems can be further optimized for targeted and efficient drug delivery. A promising direction involves the incorporation of functional features that enhance the nanocarrier's ability to localize at diseased tissues, regulate therapeutic release, and engage dynamically with the pathological microenvironment. These functional strategies, including surface modifications to improve tissue targeting and structural adaptations to enable controlled and responsive drug release, play a pivotal role in translating nanomedicine concepts into viable therapeutic platforms. The following sections will provide a detailed overview of these functional strategies and their potential to enhance the precision and therapeutic performance of nanocarrier-based interventions for gout.

### 3.4 Functionalization strategies in nanocarrier in the treatment of gout

This potential is demonstrated in nanocarrier systems designed for gout treatment, where the release of active agents is guided by physiological triggers such as pH changes, oxidative stress, and polymer degradation. These smart systems adapt to the inflammatory environment to improve drug solubility, enhance targeting, and ensure a more controlled therapeutic effect. Reactive oxygen species (ROS)-responsive nanocarrier systems have emerged as promising strategies for targeted drug delivery, especially in inflammatory conditions such as gout, where elevated ROS levels are characteristic of the diseased microenvironment. The polydopamine-platinum nanocarrier treats acute gout through a multi-step process that responds to the unique microenvironment of the affected tissues, as discussed in the paper "Mild hyperthermia-enhanced synergistic uric acid degradation and multiple ROS elimination for effective acute gout therapy” (Zhao et al., 2024[126]). It begins with the formation of polydopamine nanoparticles, which are created by allowing dopamine to self-polymerize in an alkaline solution. These nanoparticles serve as a platform for platinum to bind and grow, resulting in a stable core-shell structure with a large surface area and plenty of reactive sites for further interaction. The platinum particles act like several natural enzymes, mimicking the activity of uricase, catalase, and superoxide dismutase. This allows the system to break down uric acid into more soluble forms while eliminating harmful reactive oxygen species, such as superoxide anions, hydrogen peroxide, and hydroxyl radicals. Unlike natural uricase, this nanocarrier avoids producing hydrogen peroxide during uric acid degradation, which helps prevent further inflammation. Meanwhile, the polydopamine itself plays a supportive role by neutralizing reactive oxygen species and helping to repair damaged cells. Both polydopamine and platinum are also capable of absorbing Near-Infrared-II (NIR-II) light, which allows the system to gently heat up in response to laser exposure. This mild warming effect enhances the enzyme-like activity of the nanocarrier, improves uric acid breakdown, increases oxygen levels, and helps to alleviate the hypoxic conditions often present during gout inflammation. It also improves the solubility of MSU crystals, making them easier to metabolize and clear. In addition, this system helps reduce inflammation by lowering the activity of the NF-κB signaling pathway, which in turn reduces the release of inflammatory markers like TNF-α, IL-6, and IL-1β. By combining multiple therapeutic effects such as uric acid degradation, reduction of oxidative stress, alleviation of hypoxia, suppression of inflammation, and promotion of cellular recovery, this nanomedicine presents a comprehensive and promising approach for the treatment of acute gout.

Building upon the discussion of nanocarrier systems, it is important to consider how polymer degradation influences drug release behavior. The drug release mechanism of poly(lactic-co-glycolic acid) (PLGA) nanoparticles follows a multi-phase degradation process that ensures sustained and controlled therapeutic effects, especially useful in treating conditions like gout. The first phase involves an initial burst release, which occurs rapidly due to the immediate diffusion of drug molecules that are loosely associated with or adsorbed on the surface of the nanoparticles. This is followed by a slower second phase driven by polymer chain scission, where the long chains of PLGA begin to break into shorter fragments, gradually releasing the encapsulated drug. Over time, the process transitions into a third and final phase characterized by bulk polymer erosion. During this stage, the structural integrity of the nanoparticle is lost, and the remaining drug is released in a sustained manner. This prolonged release is particularly beneficial for delivering uricase, a therapeutic enzyme with a naturally short half-life in the bloodstream, as it helps maintain its therapeutic levels over an extended period. When combined with anti-inflammatory agents like aceclofenac, which is also encapsulated within the nanoparticles, the system provides a dual-action effect: degrading monosodium urate crystals while simultaneously reducing inflammation. Through this controlled and time-dependent degradation of the polymer matrix, PLGA nanoparticles offer an effective and sustained therapeutic strategy for the complete resolution of urate deposits and inflammation in gout treatment, as demonstrated in the study on nanoparticulate combination therapy for urate crystal degradation (Tiwari et al., 2015[108]).

The release of allopurinol from pH-sensitive nanocarriers offers an effective strategy to improve its bioavailability and therapeutic outcome in gout treatment. These hydrogel beads are made by combining carrageenan, a naturally occurring sulfated polysaccharide, with collagen extracted from fish scales as discussed in (Nguyen et al., 2020[69]). The two components interact through ionic and hydrogen bonding, creating a stable and responsive network that reacts to changes in environmental pH. Allopurinol is incorporated into this structure and further stabilized through hydrogen bonding with the functional groups on carrageenan and fish scale collagen, which helps regulate its release and improves its solubility in biological fluids. The responsiveness of the hydrogel beads to pH is a key feature of this system. In acidic environments, the amine groups of allopurinol molecules become protonated, making the drug more dispersible and allowing it to diffuse more easily through the gel network. This pH-triggered behavior plays an important role in facilitating the release of the drug when needed. Interestingly, even though allopurinol itself tends to release better in acidic conditions, the hydrogel beads showed a higher release at physiological pH, around 7.4. This suggests that the hydrogel matrix is capable of fine-tuning the drug release based on the surrounding pH, which is especially useful in targeting specific tissues. One of the major challenges with using allopurinol is its poor solubility in acidic and aqueous environments, which can limit how well the drug is absorbed and used by the body. This hydrogel-based delivery system helps overcome that limitation by enhancing its dissolution rate, with studies showing a 1.6 to 6.7 times increase in drug release compared to its crystalline form. This improvement allows for more consistent and extended drug delivery over time, which is important for maintaining effective treatment levels in the body. By offering a controlled and sustained release, this system has the potential to reduce the number of doses required and improve patient compliance, ultimately making gout management more effective and convenient.

### 3.5 Integrating co-encapsulation with sequential release

Co-encapsulation and sequential release are innovative ways to distribute drugs that attempt to improve their effectiveness, optimize their release profiles, and reduce their systemic side effects. These methods are especially helpful for treating complicated disorders that need more than one drug to be given at the same time or in a planned order. Both techniques are widely used together in multifunctional delivery systems to meet a wide range of therapeutic needs, even though they have different mechanistic focusses: co-encapsulation focusses on simultaneous administration, whereas sequential release focusses on temporal control.

Co-encapsulation is when two or more active pharmaceutical ingredients are put into the same carrier system so that they can be delivered to the same target site at the same time. This method works well for therapies where medications work better together or need to be kept stable while they move through biological settings. One example is putting Vitamin C and GX-50 together in liposomes, where Vc was in the water compartment and GX-50 was in the lipid bilayer (Wang et al., 2024[115]). This separation made both chemicals more chemically stable and better at treating diseases. Compared to formulations with only one drug, the resulting system had better antioxidant activity and tyrosinase inhibition. The release behavior followed the Korsmeyer-Peppas model, which showed that both diffusion and matrix erosion controlled the rate at which the drug was released. These are important for getting sustained or delayed release profiles.

**Figure 5:**
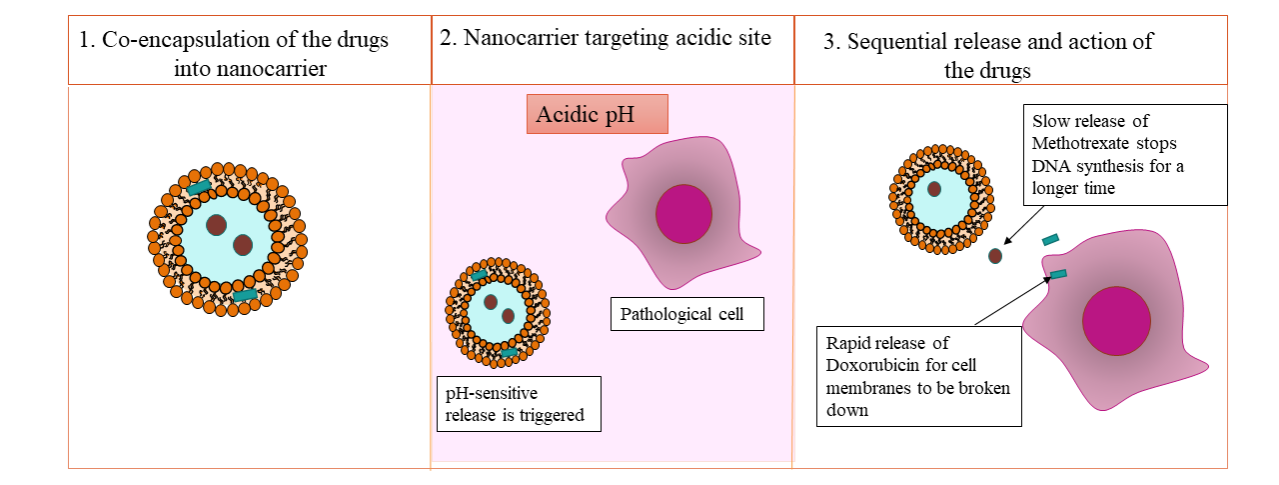
Design of a pH-triggered co-delivery system for sequential doxorubicin and methotrexate release

One interesting case was demonstrated in Figure 5(Fig. 5) the use of pH-responsive chitosan/alginate nanogel nanocarriers to transport both doxorubicin and methotrexate at the same time, taking advantage of the acidic cancer microenvironment (Costa-e-Sá et al., 2024[19]). In this method, doxorubicin was designed to be released quickly in acidic circumstances, which allowed cancer cell membranes to be broken down early. Methotrexate, on the other hand, was released more slowly, which stopped DNA synthesis for a longer time. This separation in time, made possible by the smart design of the carrier's pH-sensitivity, showed improved therapeutic synergy by matching the drug's activity with the tumor's pathological evolution.

In the same way, fucoxanthin (FUC) and curcumin (Cur) were put together in a solid-in-oil-in-water (S/O/W) emulsion with each drug carefully placed in a different section of the formulation (Wang et al., 2024[113]). This design made it possible to manage the release of the two compounds in space and time. One molecule was released early in the upper gastrointestinal system, while the other was kept safe for release later in the digestive tract. These examples show how functionalization can be used to synchronise co-delivery and phase-specific release.

On the other hand, sequential release is meant to administer several agents in a regulated way that depends on time. This kind of time control is especially important in treatments where the effects of drugs only happen at certain stages or in a certain order. In a trial with multilayer film devices, the order in which pirfenidone and ketoprofen were released could be exactly controlled by changing the position of the drugs and the amount of plasticiser (Jennings et al., 2017[43]). The outcome was a planned release pattern that kept working for several days to reduce inflammation and fibrosis. Another design used "forward" and "reverse" layering methods to control when the drug was released dependent on the composition of the matrix. This showed that drug activation can be changed.

It is also important to note that some delivery methods are made just for sequential distribution without co-encapsulation. In these systems, medications are put into several phases or layers, and the timing of their release is controlled by how quickly the carrier breaks down or how quickly it spreads. Even though these kinds of systems do not entail co-localization within a single matrix, they still let drugs work in ways that are specific to biological demands. To sum up, co-encapsulation and sequential release are not mutually exclusive in the field of current drug delivery science. Instead, they work hand-in-hand. By combining them into one platform, numerous medicines can be delivered at the same time with site-specific and temporally controlled release. This improves therapeutic performance while lowering toxicity. These technologies are a huge step forward in the development of next-generation treatments and could be very useful in personalized medicine.

### 3.6 Co-encapsulation opportunities in gout treatment

These kinds of new ideas are a great way to move forward with the creation of personalized and very effective therapeutic systems. Advanced nanocarrier-based formulations have the potential to help manage complex diseases by customising drug delivery to the unique pharmacokinetic and therapeutic needs of different agents. This is especially true for diseases like gout, which need both long-term urate control and quick action during acute flares. In theory, co-encapsulation and sequential release techniques could meet both of these needs by allowing anti-inflammatory and urate-lowering medicines to be released at the same time or at different times within a single nanocarrier system. This might make it easier to take pills, make people more likely to follow the rules, and make drugs work better in inflamed joints or throughout the body.

When choosing medications to co-encapsulate with allopurinol or febuxostat, the formulation strategy must be carefully linked with the therapeutic goals, which could be to reduce urate levels, treat acute inflammation, or do both in a dual-action regimen. Meloxicam and etoricoxib stand out among NSAIDs as theoretically good candidates for co-formulation with either urate-lowering agent because they have a long half-life, continue to work as anti-inflammatories, and do not cause any severe metabolic or transporter-mediated problems. There have been no reports of co-delivery systems like this being used to treat gout in the clinic. However, putting allopurinol-meloxicam or febuxostat-meloxicam in long-acting carriers like PEGylated liposomes or niosomes may help the drugs work longer and make patients more likely to stick to their treatment, especially for long-term therapy.

In acute-phase applications, prednisolone might conceivably be used as a co-encapsulation partner for allopurinol or febuxostat because it starts working quickly, suppresses the immune system, and does not interfere much with important drug-metabolizing enzymes or transporters. Fast-release nanocarrier might be made to give prednisolone and a urate-lowering drug at the same time, which would be helpful during flare-ups. The pharmacokinetics of allopurinol and prednisolone are compatible since prednisolone has a short half-life and is cleared by the kidneys. Febuxostat's hepatic elimination profile does not provide any known interaction risk, therefore both combinations are worth looking into in the future.

Another drug that could be used with other drugs is diclofenac. Its ability to quickly relieve pain and penetrate synovial fluid make it useful for localized administration systems such intra-articular injectables or transdermal patches when used with allopurinol or febuxostat. There have been no direct metabolic problems seen with the xanthine oxidase inhibitors, even though they are broken down by several CYP enzymes. If the renal effects are well managed, giving diclofenac with either medicine may provide further benefits.

Because it has a long half-life and targets microtubules, colchicine should also be considered for inclusion in sustained-release co-encapsulation platforms. There may be worries about co-delivery with allopurinol because they both use the same renal elimination pathways. However, careful dose planning or separating the two drugs in time within the carrier system could lower the risk of toxicity. Co-formulation with febuxostat seems better since febuxostat does not change the activities of CYP3A4 or Pgp, which are important for managing colchicine. This means that there is a decreased chance of pharmacokinetic interaction.

Short-acting NSAIDs, such as ibuprofen and naproxen, may not be the best choice for long-term use, but they could work in rapid-release co-delivery systems made for quick symptom relief. They do not interact much with xanthine oxidase inhibitors, and their pharmacokinetics are predictable, therefore they are relatively safe theoretical possibilities for combination formulations.

Celecoxib, on the other hand, could be difficult. It seems that febuxostat is compatible with allopurinol in terms of pharmacokinetics. However, febuxostat's recognized ability to block the breast cancer resistant protein (BCRP), which is a transporter that helps get rid of celecoxib, enhances the risk of higher systemic exposure and related side effects. If this combination were to be looked at further, it would probably need careful dose adjustment or compartmentalized distribution to be safe.

Overall, putting urate-lowering medications like allopurinol or febuxostat together with certain anti-inflammatory drugs is a potentially appealing way to manage gout in a personalized way. Even if it's still in the preclinical stage, this method lets you change the release kinetics, target inflamed or metabolically active areas, and lower systemic toxicity by using nanocarrier design. As research moves forward, it will be very important to pay close attention to medication compatibility, vesicle stability, and clinical validation in order to turn these combinations from promising ideas into real treatments.

## Conclusion

Gout continues to impose a substantial clinical burden, driven by the intricate interplay between persistent hyperuricemia and episodic, yet debilitating, inflammation. While conventional monotherapies remain foundational, their limitations such as inadequate dual-action control, inconsistent pharmacokinetics, and safety concerns underscore the urgent need for more sophisticated therapeutic strategies. Nanocarrier-based drug delivery systems emerge as a promising paradigm shift, enabling precise co-delivery of urate-lowering and anti-inflammatory agents with enhanced bioavailability and reduced systemic toxicity. The integration of advanced functionalization techniques further empowers these platforms to respond intelligently to the inflamed joint microenvironment, particularly to acidic pH and oxidative stress. Such dual-responsive nanocarriers represent a significant leap toward personalized and disease-targeted interventions. Although clinical translation is still evolving, the compelling preclinical evidence highlights their transformative potential in redefining the therapeutic landscape of gout. Continued interdisciplinary research and early-phase clinical trials will be pivotal in bridging the gap between bench and bedside, ultimately advancing precision medicine in gout management.

## Notes

Syed Mahmood and Noraini Ahmad (Department of Chemistry, Faculty of Science, Universiti Malaya, 50603 Kuala Lumpur, Malaysia; E-mail: ainie@um.edu.my) contributed equally as corresponding author.

## Declaration

### Acknowledgments

The authors gratefully acknowledge Universiti Malaya (UM) for the financial support provided through the UM Research Fund Assistance (BKP Special) (BKS001-2024). The authors also extend their sincere appreciation to the Ministry of Education, Culture, Research, and Technology of Indonesia, through the Indonesia Endowment Funds for Education (LPDP) under the Enhancing Quality Education for International University Recognition (EQUITY) program, hereafter referred to as the Higher Education Endowment Fund Program (DAPT) via the ITS Inbound Researcher Mobility 2023 scheme. The authors further express their gratitude to the Faculty of Medicine and Health, Institut Teknologi Sepuluh Nopember, and the Faculty of Dentistry, Universitas Airlangga, Surabaya, for their valuable research collaboration and support.

### Conflict of interest

The authors declare no conflict of interest.

### Artificial Intelligence (AI) - Assisted Technology

No AI tool was used for the write-up of the manuscript.

### Author contributions: CRediT

Nisha Rata Karusan: Writing - original draft

Hairul Anuar Tajuddin: Supervision; Writing - review and editing

Nor Azlin Mat Radi: Writing - review and editing

Rumman Karimah: Writing - review and editing

Pratiwi Soesilawati: Writing - review and editing

Syed Mahmood: Supervision; Methodology; Writing - review and editing

Noraini Ahmad: Conceptualization; Funding acquisition; Supervision, Writing - review and editing

## Figures and Tables

**Table 1:**
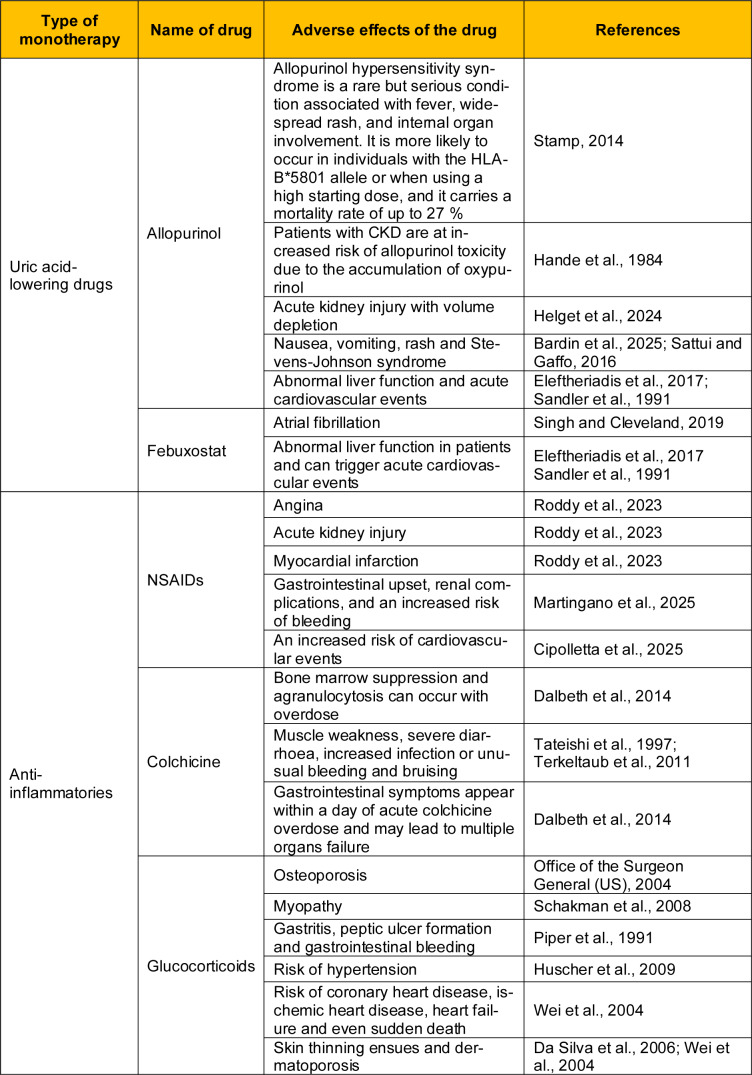
Summary of adverse events related to conventional monotherapies for gout treatment

**Table 2:**
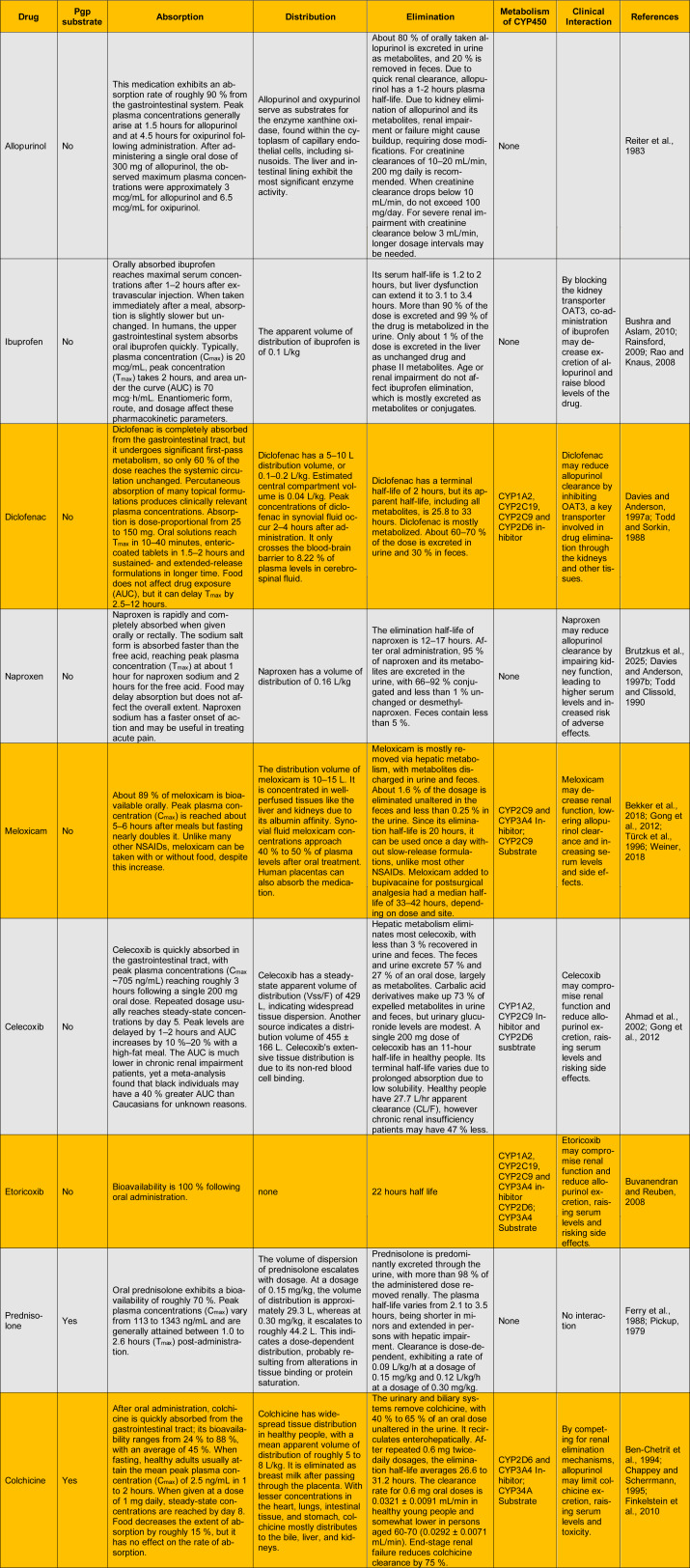
Overview of pharmacokinetic parameters and potential interaction risks between allopurinol and gout-associated medications

**Table 3:**
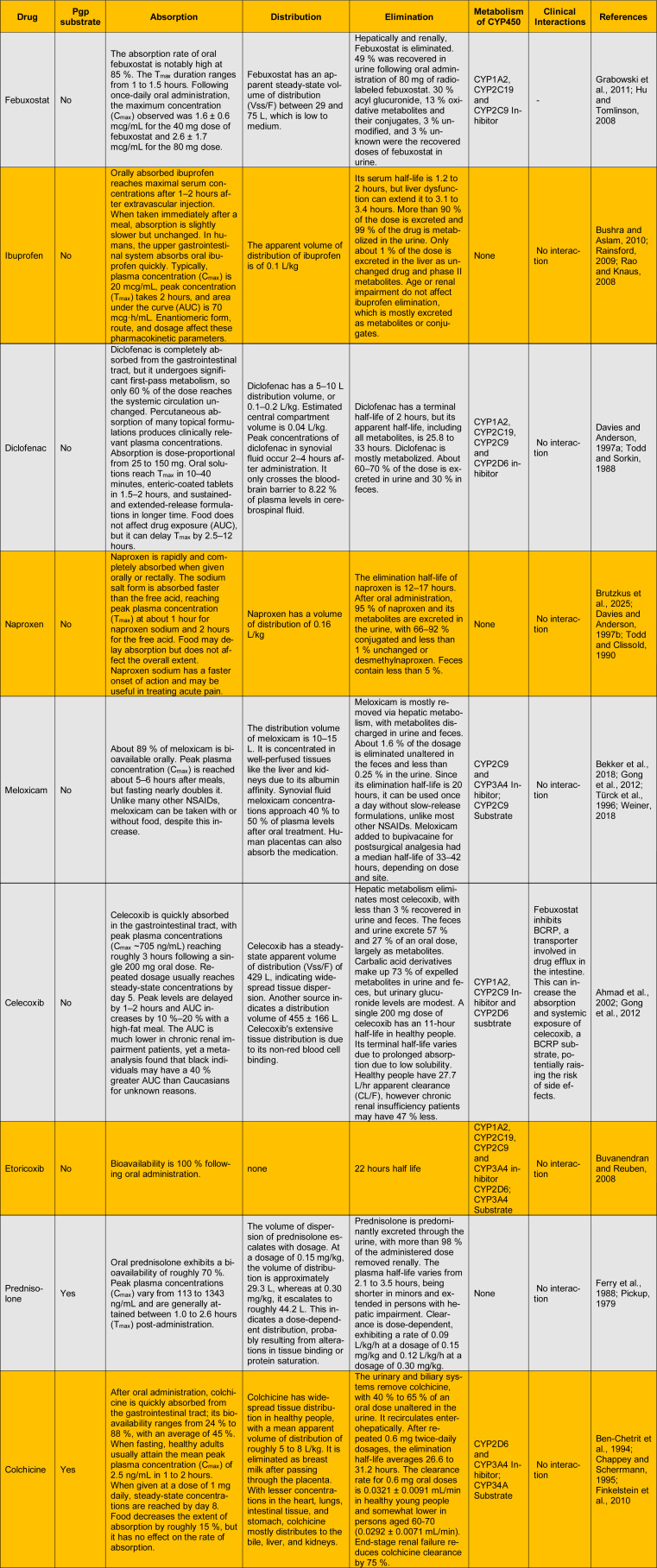
Overview of pharmacokinetic parameters and potential interaction risks between febuxostat and gout-associated medications.

**Figure 1:**
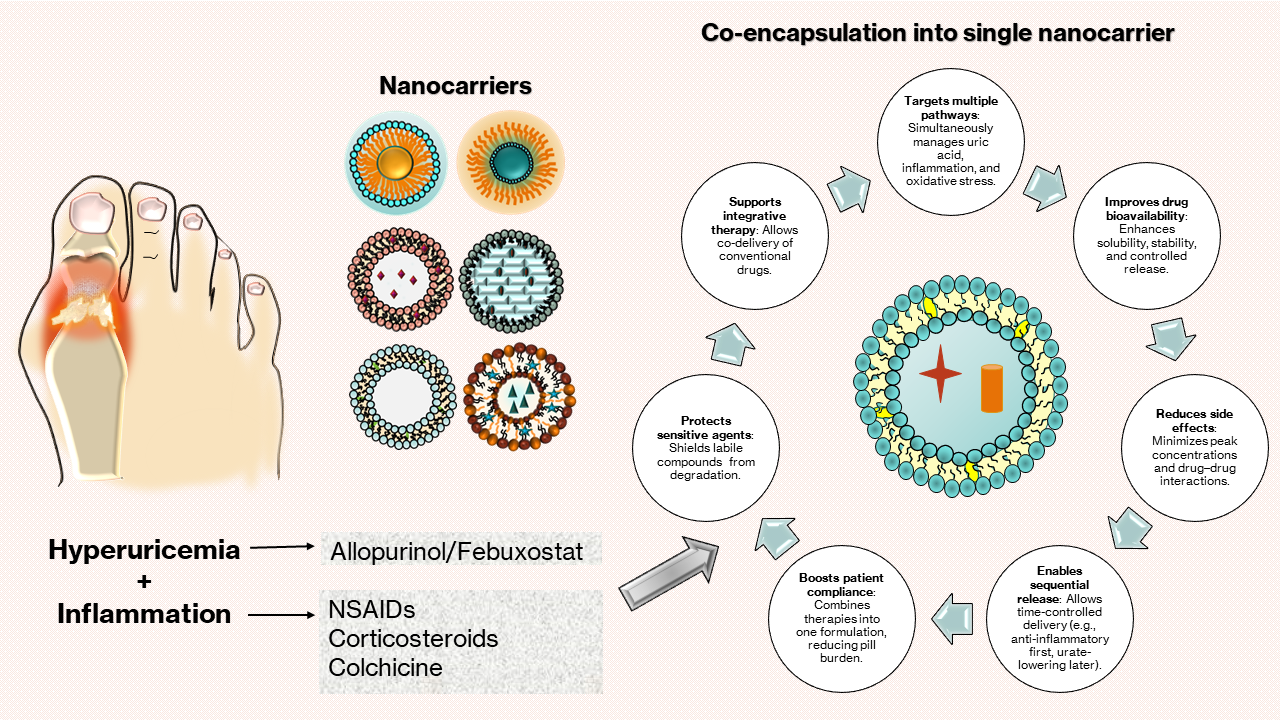
Graphical abstract
